# ^18^F‐ FDG PET Reveals a Nucleus Accumbens‐Centered Metabolic Network Correlating With Clinical Severity in Anti‐LGI1 Encephalitis

**DOI:** 10.1002/mco2.70544

**Published:** 2025-12-14

**Authors:** Binbin Nie, Xuan Xu, Wenyue Dong, Leilei Yuan, Hengri Cong, Yueta Ma, Huabing Wang, De‐Cai Tian, Linlin Yin, Tian Song, Yanxue Zhao, Guoqiang Chang, TianJie Lyu, Yun Liu, Wenping Ma, Fu‐Dong Shi, Lin Ai, Wangshu Xu

**Affiliations:** ^1^ Beijing Engineering Research Center of Radiographic Techniques and Equipment, Institute of High Energy Physics Chinese Academy of Sciences Beijing China; ^2^ School of Nuclear Science and Technology University of Chinese Academy of Sciences Beijing China; ^3^ Department of Neurology Beijing Tiantan Hospital Capital Medical University Beijing China; ^4^ Peking University Third Hospital Beijing China; ^5^ Department of Nuclear Medicine Beijing Tiantan Hospital Capital Medical University Beijing China; ^6^ Department of Neurology Tianjin Medical University General Hospital Tianjin China; ^7^ Department of Neurosurgery Beijing Children's Hospital, Capital Medical University, National Center for Children's Health Beijing China

**Keywords:** anti‐LGI1 encephalitis, degree of disease severity, metabolic covariation, PET

## Abstract

The metabolic signature of anti‐leucine‐rich glioma‐inactivated 1 (anti‐LGI1) autoimmune encephalitis remains poorly defined. We sought to delineate disease‐specific ^18^F‐FDG PET patterns and assess their relationships with clinical severity and cognition. Forty‐seven patients with anti‐LGI1 encephalitis and 25 healthy controls underwent ^18^F‐FDG PET/CT, and voxel‐wise comprised to identify regional metabolic alterations. A disease‐specific metabolic pattern was derived with fivefold cross‐validation, and a metabolic covariance network was mapped using the Brainnetome atlas. Pattern expression scores were correlated with clinical assessments. Compared to controls, patients demonstrated hypermetabolism in the hippocampal rostal, nucleus accumbens (NAc), and hypothalamus, alongside hypometabolism in the dorsolateral prefrontal cortex and posterior cingulate cortex (PCC). We identified a robust metabolic pattern centered on the NAc with extensions to the hippocampus, prefrontal cortex, and PCC; expression of this pattern correlated positively with both clinical severity and cognitive impairment. Subgroup analyses showed no significant differences in basal ganglia metabolism between patients with and without faciobrachial dystonic seizures (FBDS), or in hypothalamic metabolism between those with and without hyponatremia. Overall, ^18^F‐FDG PET uncovers a NAc‐centered metabolic network that parallels disease severity in anti‐LGI1 encephalitis. Our study offers potential biomarker for clinical evaluation and provides valuable insights into the underlying pathogenesis of clinical manifestations.

## Graphical abstract

This study provides a comprehensive ^18^F‐FDG PET‐based assessment of whole‐brain metabolism in the acute phase of anti‐LGI1 encephalitis. We identified a covariant metabolic network originating in the NAc; the strength of this network correlated positively with both clinical severity and cognitive impairment. This pattern offers potential biomarker for clinical evaluation and valuable insights into the underlying pathogenesis of clinical manifestations.

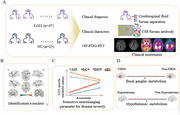

## 1 Introduction

Anti‐leucine‐rich glioma inactivated 1 (anti‐LGI1) autoimmune encephalitis is a rare subtype of encephalitis that usually manifests as limbic encephalitis [1, 2]. Patients with anti‐LGI1 encephalitis may exhibit persistent cognitive decline [3], psychobehavioral abnormalities [4], and mood disorders, including anxiety and depression [5], seizures [6], refractory hyponatremia, and other symptoms. Magnetic resonance imaging (MRI) frequently reveals T2‐weighted and/or fluid‐attenuated inversion‐recovery (FLAIR) hyperintensities in the basal ganglia (BG) and medial temporal lobes [6, 7]. Notably, up to 36% of patients lack signal abnormalities in these regions, yet exhibit metabolic changes that are readily detectable with 2‐deoxy‐2‐[fluorine‐18]fluoro‐D‐glucose integrated with computed tomography (^18^F‐FDG PET/CT) [8].

Accumulating evidence suggests that ^18^F‐FDG PET/CT outperforms MRI for detecting acute anti‐LGI1 encephalitis [9]. Hypermetabolism of the BG and medial temporal lobe is a consistent PET finding [10, 11, 12], although the precise sub‐regional distribution of these changes remains to be clarified [13, 14]. Focal hypometabolism, particularly in the prefrontal cortex (PFC), has also been reported [9, 10], but how hyper‐ and hypometabolic patterns interact at a subregional level is still poorly understood.

Clinically, anti‐LGI1 encephalitis often presents with persistent long‐term memory impairment during the recovery phase [3], which has been linked to hippocampal metabolic abnormalities. However, it remains unclear whether other brain regions are involved in the disease process. Additionally, the acute phase of anti‐LGI1 encephalitis is the first to show characteristic FBDS and hyponatremia [15]. These clinical manifestations were thought to be associated with dysfunction of the basal ganglia and hypothalamus (HYP), although there have been no specific studies of metabolism in these two regions [16, 17]. Early recognition of FBDS through metabolic manifestations can help in early diagnosis.

Therefore, this study aims to characterize the cerebral metabolic patterns and specific subgroups in anti‐LGI1 encephalitis using ^18^F‐FDG PET imaging, and to investigate their associations with clinical manifestation. By proposing a novel metabolic pattern, our findings may provide imaging‐based evidence to support the clinical utility of PET in anti‐LGI1 encephalitis and offer new insights into its underlying pathophysiological mechanisms.

## 2 Results

### 2.1 Clinical Manifestations

**FIGURE 1:**
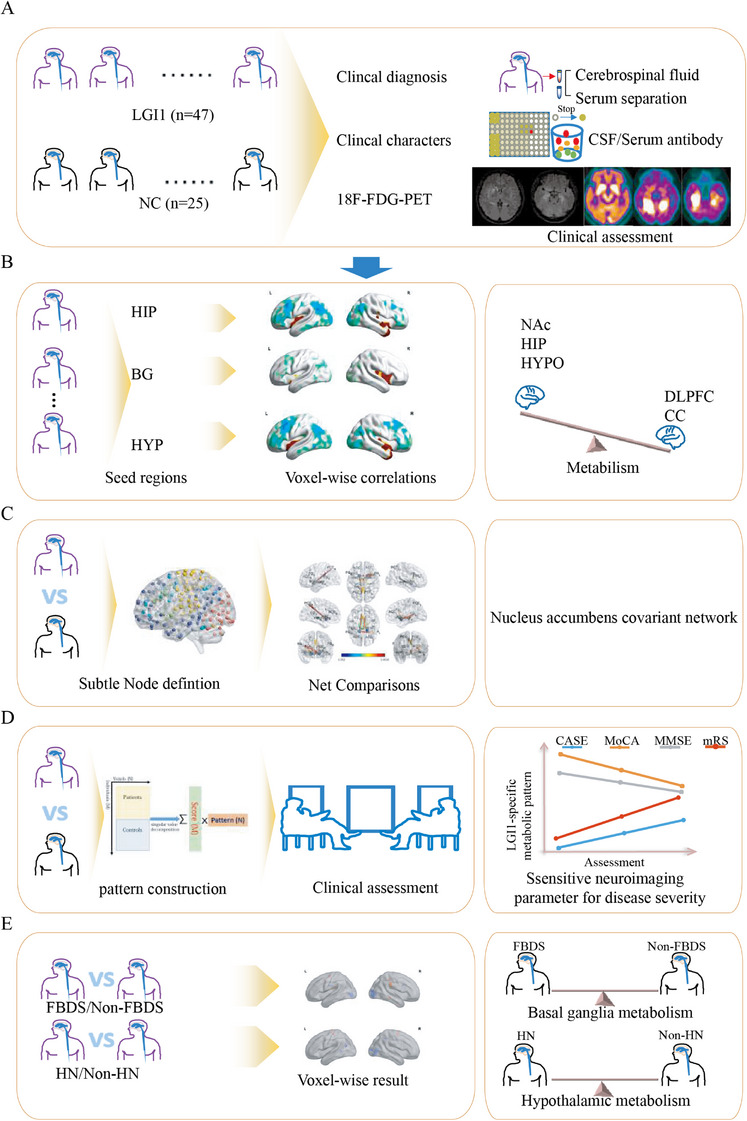
Study workflow and principal findings. (A) Forty‐seven acute anti‐LGI1 encephalitis patients and 25 controls underwent ^18^F‐FDG PET/CT. (B) Voxel‐wise sub‐regional analyses showed hypermetabolism in the rostral HIP, nucleus accumbens (NAc) and hypothalamus, and hypometabolism in the dorsolateral prefrontal cortex (DLPFC) plus both anterior and posterior cingulate cortex (ACC/PCC). (C) Metabolic covariance mapping revealed a disease‐specific network centered on the NAc and extending to the rostral HIP, DLPFC, and PCC. (D) Expression scores of this “LGI1 pattern” correlated positively with mRS and CASE scores, and negatively with MoCA and MMSE scores, indicating higher network expression reflects greater acute severity and worse cognition. (E) Basal‐ganglia metabolism did not differ between patients with and without FBDS; similarly, HYP metabolism was comparable in patients with and without hyponatraemia. ACC, anterior cingulate cortex; BG, basal ganglia; CASE, Clinical Assessment Scale for Autoimmune Encephalitis; CSF, cerebrospinal fluid; DLPFC, dorsolateral prefrontal cortex; FBDS, faciobrachial dystonic seizures; ^18^F‐FDG PET, 2‐deoxy‐2‐[fluorine‐18]fluoro‐D‐glucose integrated with computed tomography; HIP, hippocampus; HN, hyponatremia; HYP, hypothalamus; LGI1, leucine‐rich glioma inactivated 1; MMSE, Mini‐Mental State Examination; MoCA, Montreal Cognitive Assessment; MRI, magnetic resonance imaging; mRS, Modified Rankin Scale; NAc, nucleus accumben; NC, normal control; PCC, posterior cingulate cortex.

The study workflow is presented in Figure 1. Of all the enrolled patients, 47 patients fulfilled diagnostic criteria for acute anti‐LGI1 encephalitis (Table S1); three of them also underwent follow‐up ^18^F‐FDG PET/CT in the remission phase. Twenty‐five age‐matched healthy individuals served as controls (median age 59 years, inter‐quartile range [IQR]: 20 years). Within the patient cohort, 24 (51%) experienced FBDS and an equal number presented with hyponatremia. Seizures of any type were documented in 42 patients (89%). Cognitive decline was noted in 33 (70%), reduced consciousness in eight (17%), and psychobehavioral disturbances in five (11%). Thyroid dysfunction was present in 23 (49%) cases. No patient had an associated neoplasm (Table 1).

**TABLE 1:** The clinical characteristics of patients with anti‐LGI1 encephalitis in acute phase *n* (%).

Characteristics	Values
Age	59 (IQR:20)
Sex	
Male	25 (53%)
Female	22 (47%)
Acute phase	47
Clinical symptoms	
FBDS	24 (51%)
Hyponatremia	24 (51%)
Seizures	42 (89%)
Cognitive decline	33 (70%)
Loss of consciousness	8 (17%)
Psychobehavioral abnormality	15 (32%)
Thyroid dysfunction	23 (49%)
Tumors	0 (0%)
MRI abnormalities	32 (68%)
Medial temporal lobe abnormalities	27 (57%)
Basal ganglia abnormalities	6 (13%)
Medial temporal lobe and Basal ganglia	1 (2%)

Abbreviations: FBDS, faciobrachial dystonic seizures; IQR, interquartile range; MRI, magnetic resonance imaging.

### 2.2 ^18^F‐FDG PET Metabolic Pattern

**FIGURE 2:**
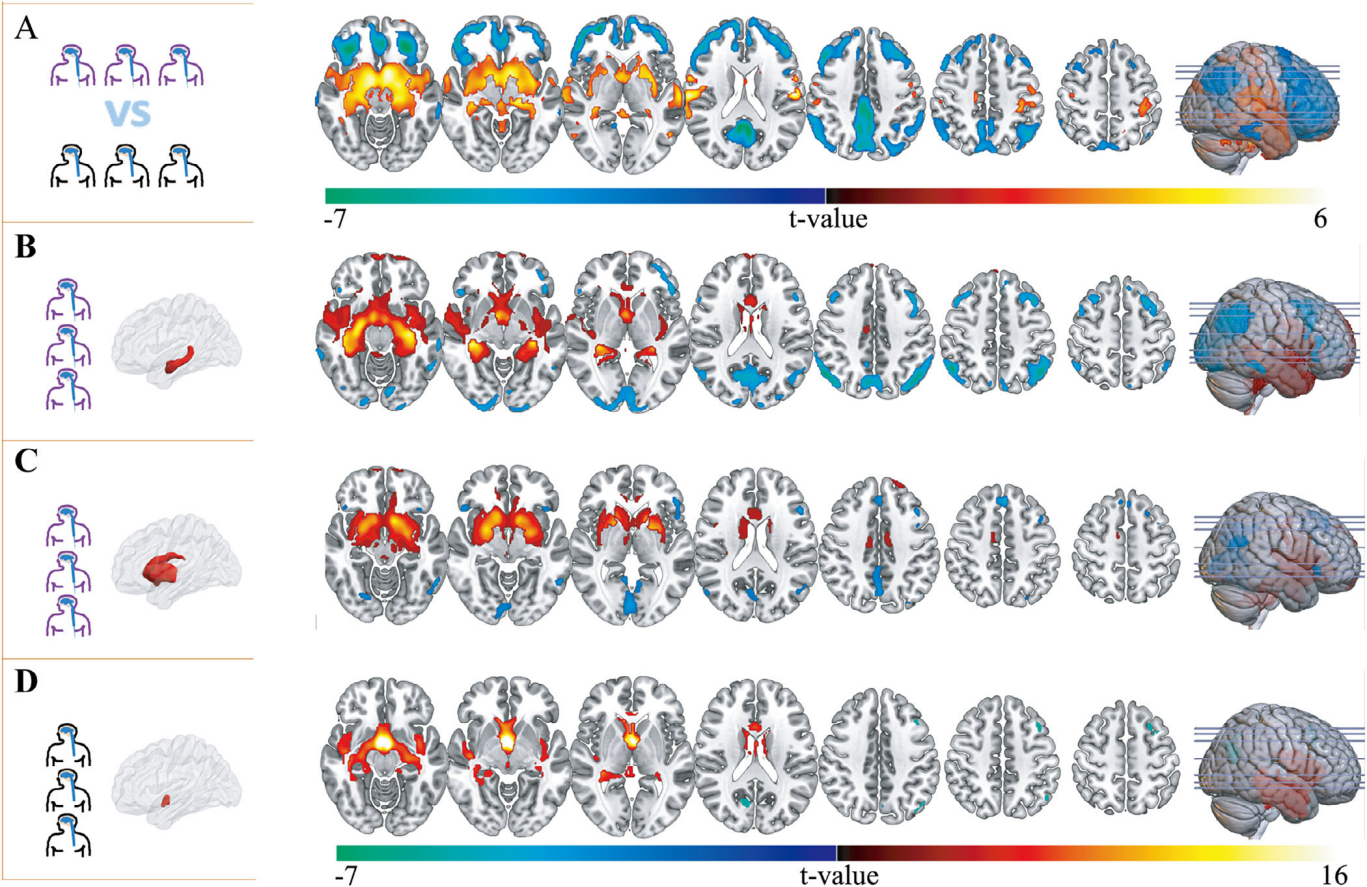
Voxel‐wise metabolic alterations and covariance networks. (A) Statistical parametric map comparing anti‐LGI1 encephalitis (*n* =  47) with controls (*n* =  25). Hot colors denote hypermetabolism; cool colors denote hypometabolism. Threshold: voxel‐wise *p* < 0.001, cluster‐level GRF‐corrected *p* < 0.05. (B–D) Seed‐to‐whole‐brain correlation maps for the hippocampus (B), basal ganglia (C), and hypothalamus (D), respectively (voxel‐wise *p* < 0.001, cluster‐level GRF‐corrected *p* < 0.05). Warm colors indicate positive correlations; cool colors indicate negative correlations. The color bar represents *t*‐values. SPM, statistical parametric mapping.

In the acute phase, voxel‐wise ^18^F‐FDG PET analysis showed that patients with anti‐LGI1 encephalitis had significantly increased glucose metabolism in the hippocampus (HIP), BG, and HYP, whereas marked hypometabolism was evident in the DLPFC, precuneus (PCUN), and cingulate cortex (CC) when compared with healthy controls (Figure 2A; Figure S1). Metabolic covariance mapping revealed a coherent network linking these regions. Hippocampal uptake correlated positively with BG activity and negatively with metabolism in the prefrontal, PCUN, and CC regions (Figure 2B). Likewise, elevated BG metabolism was associated with reduced uptake in the prefrontal, PCUN, and CC, and with heightened hippocampal activity (Figure 2C). The HYP displayed a positive association with medial‐temporal metabolism and an inverse relationship with the cuneus (Figure 2D). Collectively, these findings indicate that the BG‐centered metabolic network exhibits the strongest inter‐regional coupling, followed by HIP‐ and HYP‐centered connections.

### 2.3 Subregional Metabolism in Anti‐LGI1 Encephalitis Patients

**FIGURE 3:**
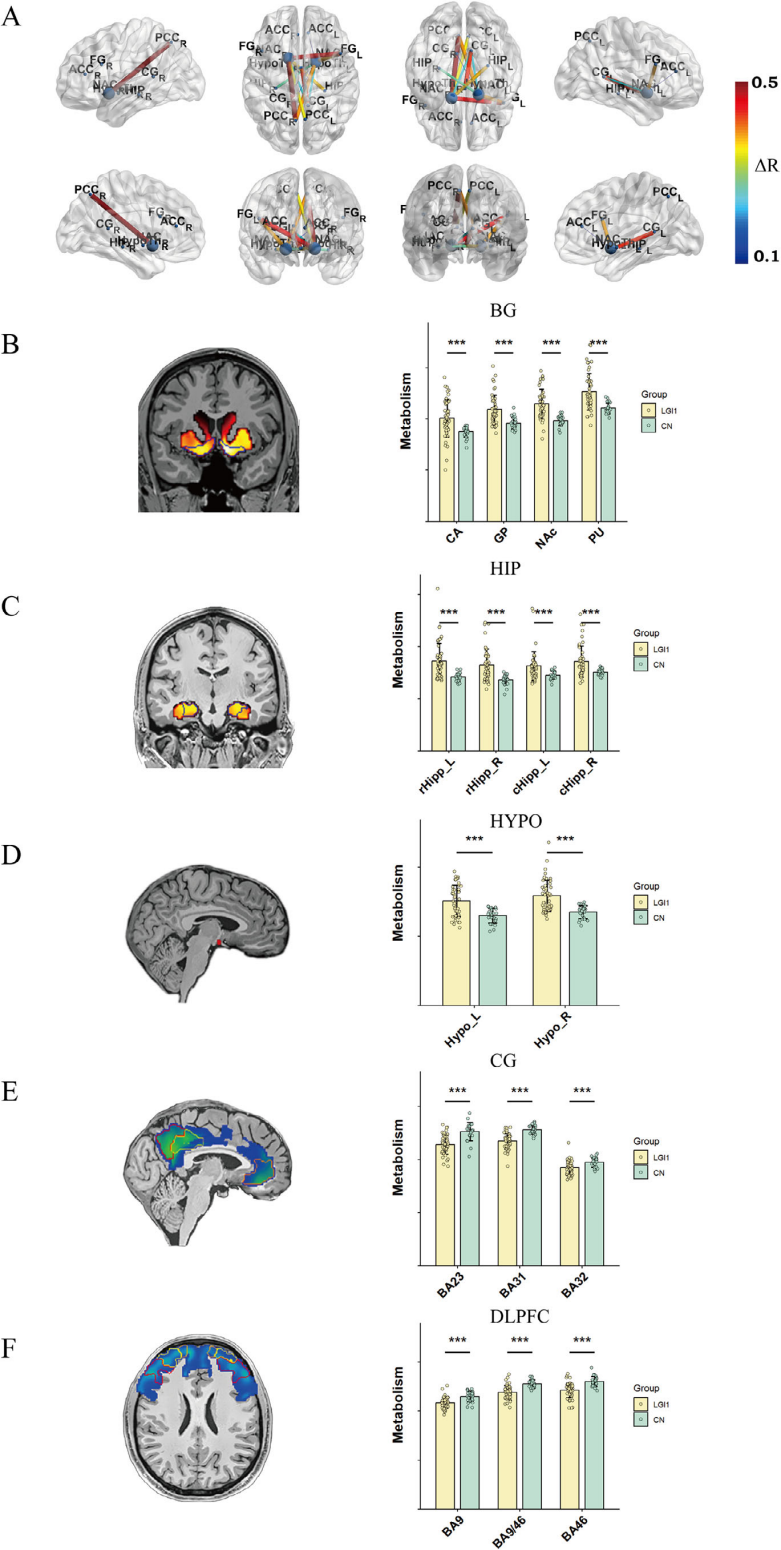
Sub‐regional metabolic network and SUVR changes. (A) Permutation testing (5000 iterations) identified a disease‐specific ^18^F‐FDG metabolic connectivity network centered on the NAc. The color bar represents the absolute difference (ΔR) in pairwise connectivity between patients and controls. (B–F) Box‐and‐whisker plots of SUVR for the regions of interest: (B) cingulate cortex (CC), (C) basal ganglia (BG), (D) hippocampus (HIP), (E) hypothalamus (HYP), and (F) dorsolateral prefrontal lobe (DLPFC). ***p < 0.001. NC, normal control.

A discrete metabolic covariance network anchored in the nucleus accumbens (NAc) and extending to the rostral HIP, DLPFC, and PCC emerged as a prominent disease‑specific feature (Figure 3A). U Sub‐regional quantification showed the largest standardized‐uptake‐value ratio (SUVR) deviations within the BG, namely, the caudate nucleus (CA), globus pallidus (GP), NAc, putamen (PU) of the BG (Figure 3B, Figure S2B), and within the HIP, where both rostral (rHipp_L, rHipp_R) and caudal (cHipp_L, cHipp_R) segments were significantly altered (Figure 3C). Additional abnormalities were detected in the bilateral HYP (Figure 3C), the BA23, BA31, and BA32 regions of the CC (Figure 3E, Figure S2C) and the BA46, BA9/46, and BA9 regions of DLPFC (Figure 3F, Figure S2A).

### 2.4 ^18^F‐FDG PET Metabolic Analysis of Two Specific Symptoms in Anti‐LGI1 Encephalitis

**FIGURE 4:**
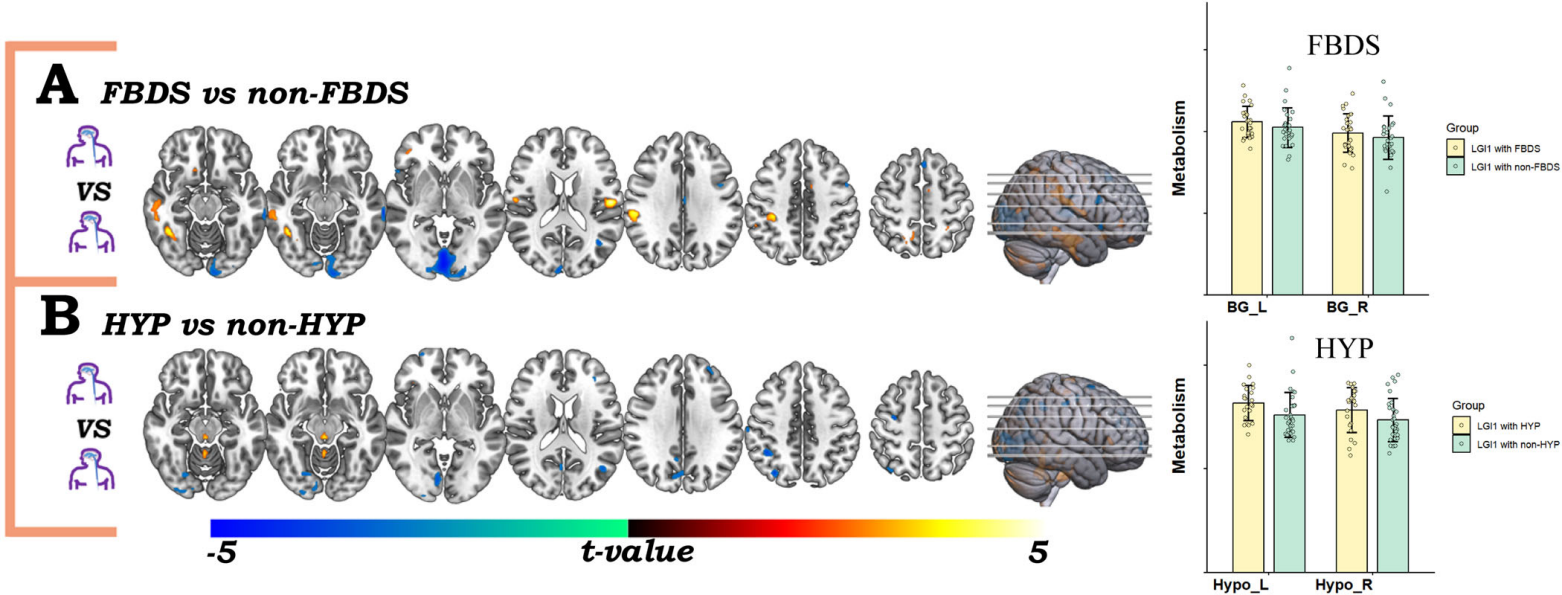
Subgroup analyses of voxel‐wise metabolic changes. (A) FBDS versus non‐FBDS group and comparison of basal ganglia metabolism. (B) Hyponatremia versus non‐hyponatremia group and comparison of hypothalamic metabolism, generated at the voxel‐threshold of *p* < 0.001 (GRF correction for cluster size with *p* < 0.05). Warm colors denote hypermetabolism, and cool colors denote hypometabolism. The color bar indicates *t*‐values. HYP, hyponatremia.

To explore metabolic correlates of FBDS, we stratified the anti‐LGI1 encephalitis patients into two subgroups based on the presence or absence of FBDS: the FBDS group and the non‐FBDS group. Voxel‐wise comparisons between these two subgroups revealed that, in comparison to the non‐FBDS group, the FBDS group displayed significantly pronounced hypometabolism in the PCUN. In contrast, no significant difference in basal‑ganglia metabolism was observed between the two subgroups (Figure 4A).

Refractory hyponatremia is a frequent complication of anti‑LGI1 encephalitis, often persisting despite conventional sodium replacement. To delineate its metabolic signature, we stratified the cohort into hyponatremia and non‐hyponatremia subgroup. Voxel‑wise comparison of SUVR maps revealed that the hyponatremia subgroup displayed significantly greater hypometabolism in the PCUN and frontal cortex than the non‑hyponatremia subgroup. In contrast, hypothalamic SUVRs did not differ significantly between the two groups (Figure 4B).

### 2.5 Metabolic Covariation in Relation to the Disease Severity and Cognitive Function

**FIGURE 5:**
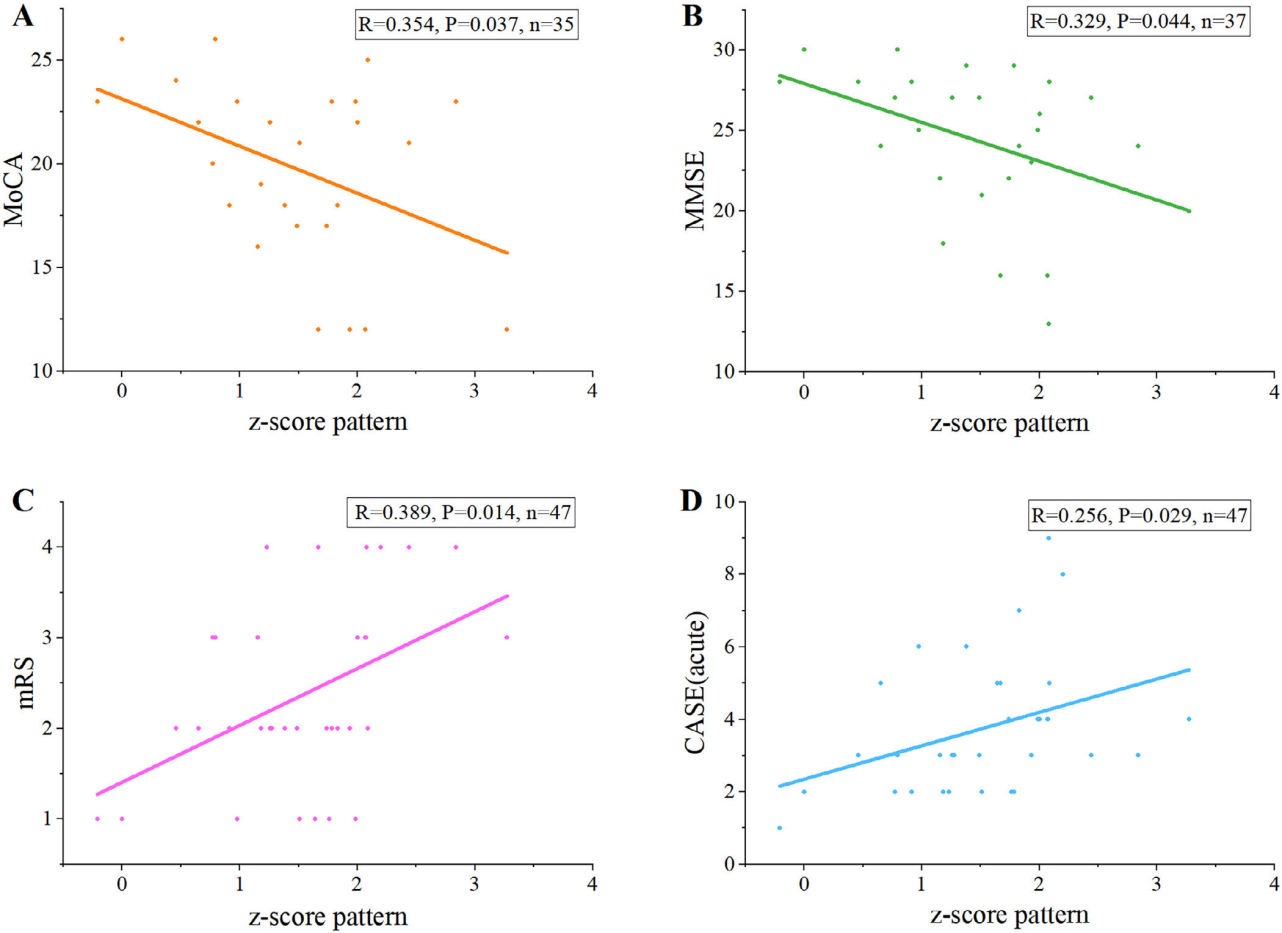
Association between LGI1‐network expression and clinical scales. (A–D) Scatter plots show correlations between the network *z*‐score and (A) MoCA, (B) MMSE, (C) mRS, and (D) CASE. CASE, Clinical Assessment Scale for Autoimmune Encephalitis; MMSE, Mini‐Mental State Examination; MoCA, Montreal Cognitive Assessment; mRS, Modified Rankin Scale.

We next explored whether expression of the LGI1‐specific metabolic pattern (network *z*‐score) relates to clinical status. Pearson's correlation was used for normally distributed variables (e.g. Montreal Cognitive Assessment [MoCA]), and Spearman's rho for non‐normal or ordinal variables (age, the Mini‐Mental State Examination [MMSE], the Clinical Assessment Scale for Autoimmune Encephalitis [CASE] and categorical variables including sex, education level, and the Modified Rankin Scale [mRS]). Interestingly, the *z*‐score correlated positively with mRS and CASE, indicating higher network expression reflects more severe neurological impairment in the acute phase. On the other hand, the *z*‐score exhibited a negative correlation with MoCA and MMSE scores, linking stronger network expression to poorer cognitive performance. However, no significant associations were found with age, sex, or years of education during the acute phase (Table 2, Figure 5). Collectively, these findings suggest that a more strongly expressed LGI1‐related metabolic network is tightly coupled to both greater acute clinical severity and worse cognitive function.

**TABLE 2:** Metabolic covariation in relation to the disease severity and cognitive function.

	MoCA^a^	Age^b^	Sex^b^	Education^b^	MMSE^b^	mRS^b^	CASE (acute)^b^	CASE (remission)^b^
Mean/median	20.29 ± 4.489	59 (IQR: 20)	—	—	25 (IQR: 7.5)	—	3 (IQR: 3)	1 (IQR: 1)
Correlation	−0.354^*^	1.36	−0.091	0.070	−0.333^*^	0.355^*^	0.318^*^	0.153
*p* (two‐tailed)	0.037	0.361	0.542	0.653	0.044	0.014	0.029	0.379
*N*	35	47	47	44	37	47	47	35

Abbreviations: CASE, Clinical Assessment Scale for Autoimmune Encephalitis; MMSE, Mini‐Mental State Examination; MoCA, Montreal Cognitive Assessment; mRS, Modified Rankin Scale.

^a^ Pearson Correlation with z‐score pattern.

^b^ Spearman Correlation with z‐score pattern.

* Correlation is significant at the 0.05 level (two‐tailed).

### 2.6 Metabolic Patterns of Patients in Remission

During follow‐up, two patients succumbed to the disease, one after intractable seizures and the other after severe psychobehavioural disturbance. Both cases had markedly elevated baseline network *z*‐scores (2.737 and 1.185, respectively), supporting the link between strong LGI1‐pattern expression and adverse outcome. All continuous variables were assessed for normality. Normally distributed data are reported as mean ± standard deviation (SD); non‑normal and categorical data are summarized as median with IQR.

Three patients underwent repeat ^18^F‑FDG PET/CT during clinical remission. In each case, the network *z*‑score declined, mirroring a reduction in basal ganglia and prefrontal covariation and a concomitant improvement in cognitive performance relative to the acute scan (Table 3). These preliminary longitudinal data further suggest that the LGI1‐specific metabolic pattern is dynamic and reversible in parallel with clinical recovery.

**TABLE 3:** Metabolic patterns of patients in remission.

	Sex	Age	*z*‐pattern	*z*‐pattern follow up
Patient 9	Female	43	0.510	−0.485
Patient 10	Female	46	1.609	1.150
Patient 14	Male	53	−0.086	−0.665

*Note*: Three patients with ^18^F‐FDG PET in acute and remission phase. z‐pattern: *z*‐score pattern in acute phase. *z*‐pattern follow up: *z*‐score pattern in remission phase.

## 3 Discussion

We conducted a comprehensive analysis of the relationship between ^18^F‐FDG‐PET metabolic patterns and the clinical manifestations and progression of anti‐LGI1 encephalitis. Using ^18^F‐FDG PET, we identified an LGI1‐specific metabolic pattern within the whole‐brain network. Specifically, we observed hypermetabolism in the BG and HIP that coincided with hypometabolism in the PFC and cuneus. Furthermore, subregional covariance analysis revealed key connections extending from the NAc to the rostral HIP, DLPFC, and PCC. This covariance‐based metabolic signature was positively associated with both disease severity and cognitive decline. Our findings suggest that anti‐LGI1 encephalitis pathogenesis may involve aberrant metabolic covariance networks underlying cognitive deficits, memory impairment, and psychiatric symptoms. Importantly, we propose that this LGI1‐specific metabolic pattern could serve as a predictive biomarker for assessing acute disease severity and cognitive function in anti‐LGI1 encephalitis, with significant implications for early detection and risk stratification.

We observed a negative correlation between the pattern *z*‐scores and MMSE and MoCA scores, indicating a close association between metabolic network alterations and cognitive dysfunction. These findings may shed light on the regional covariance mechanisms contributing to cognitive impairment [18]. Notably, during the acute phase of anti‐LGI1 encephalitis, we detected metabolic alterations in the HIP and PFC, specifically within Brodmann areas 9 and 46 (BA9/46), suggestive of structural compromise in these regions [19]. Clinically, such alterations may manifest as impaired decision‐making and executive function [20, 21, 22]. DLPFC‑mediated cognitive regulation plays a crucial role in the neural circuitry of cognitive dysfunction in acute anti‑LGI1 encephalitis [23]. Metabolic disruption within these regions may further impair memory, decision‐making, and attentional control. Previous studies have suggested that BG dysfunction exacerbates cognitive impairment via its influence on association cortices [24, 25], while hippocampal metabolic irregularities contribute to working memory [26] and episodic memory impairment [27, 28]. This complex pattern of metabolic covariance across multiple regions likely reflects widespread inflammatory involvement in anti‐LGI1 encephalitis and underlies regional functional impairments.

Moreover, we identified a core metabolic covariance network extending from the NAc to the rostral HIP, DLPFC, and PCC. The NAc, a central hub of the positive‐affect circuit [29], exhibits strong metabolic connectivity with the HIP [30], DLPFC, and insular cortex [31, 32], all integral components of the negative‐affect network [33]. This circuit underlies emotion processing [34], responses to rewarding stimuli, and reward‐punishment learning, and is closely linked to neural pathways implicated in anxiety and depression [35, 36, 37]. In anti‐LGI1 encephalitis, we posit that metabolic disturbances within the BG, a principal regulator of emotional processing, propagate through its connections to the PFC, CC, and other downstream regions, thereby contributing to deficits in cognitive control and emotional regulation and ultimately manifesting as psychiatric symptoms such as depression and irritability.

Simultaneously, we observed marked alterations in metabolic connectivity radiating from the HYP to the BG, HIP, PFC, and CC. Notably, these changes imply that the HYP may influence emotional [38], cognitive [39], and sleep‐related symptoms during the acute phase of anti‐LGI1 encephalitis via mechanisms such as behavioral regulation and sleep‐wake cycle control [40, 41, 42]. Such hypothalamic dysfunction could therefore contribute to the psychiatric manifestations characteristic of this disorder.

Importantly, the identified *z*‐score pattern correlated positively with both the mRS and the CASE score, underscoring its potential utility for risk stratification in acute anti‐LGI1 encephalitis. We also compared metabolic networks in patients with and without FBDS and hyponatremia, two hallmark clinical features of this condition [43, 44]. Earlier studies have associated FBDS with transient hypermetabolism in the BG contralateral to the seizures and in the frontal cortex [45] as well as with hypometabolism in the frontotemporal lobes [46, 47]. In our cohort of anti‐LGI1 encephalitis patients, those with and without FBDS did not differ significantly in BG or frontotemporal metabolism. Instead, we observed pronounced hypometabolism in the PCUN, which may influence motor regulation and contribute to FBDS pathophysiology in this disorder. Hyponatremia in anti‐LGI1 encephalitis is often attributed to dysregulated vasopressin secretion [48] and altered function of the hypothalamic supraoptic and paraventricular nuclei [49], yet we found no direct link between serum sodium levels and hypothalamic metabolism. Further research is warranted to clarify the hypothalamic mechanisms underlying hyponatremia in anti‐LGI1 encephalitis.

This study offers several strengths. First, by leveraging ^18^F‐FDG PET, we comprehensively characterized both global metabolic patterns and regional covariance networks in the acute phase of anti‐LGI1 encephalitis. Second, applying Brodmann area‐based parcellation allowed us to dissect subregional metabolic and connectivity alterations with high precision. Nevertheless, our analysis is limited to acute‐phase metabolic and clinical data; future investigations should incorporate longitudinal follow‐up and prognostic assessments to deepen insights into disease progression and refine clinical evaluation strategies.

## 4 Conclusion

This study provides a comprehensive ^18^F‐FDG PET‐based assessment of whole‐brain metabolism in the acute phase of anti‐LGI1 encephalitis. We delineated an LGI1‐specific metabolic signature, marked by hypermetabolism in the BG and HIP alongside hypometabolism in the PFC, cuneus, and other limbic structures. Furthermore, we identified a covariant metabolic network originating in the NAc and projecting to the rostral HIP, DLPFC, and PCC; the strength of this network correlated positively with both clinical severity and cognitive impairment. Collectively, these findings introduce a novel, noninvasive biomarker for early detection, risk stratification, and monitoring of anti‐LGI1 encephalitis, while offering fresh insights into the metabolic mechanisms underlying pathogenesis of clinical manifestations.

## 5 Materials and Methods

### 5.1 Participants

We retrospectively identified 275 consecutive patients with autoimmune encephalitis admitted to the Department of Neurology at Beijing Tiantan Hospital between January 2018 and December 2023. Of these, 92 met diagnostic criteria for anti‐LGI1 encephalitis, and 47 underwent ^18^F‐FDG PET/CT during the acute phase of their illness. Three of these patients also completed a follow‐up PET/CT during clinical remission, and their data were included in longitudinal analyses. All diagnoses adhered to the 2022 Chinese Expert Consensus on Diagnosis and Treatment of Autoimmune Encephalitis, and LGI1 autoantibodies were confirmed in serum or cerebrospinal fluid by cell‐based assay. The anti‐LGI1 cohort comprised 25 men and 22 women, with a median age of 59 years (range, 21–79 years). Demographic, clinical, and imaging data were extracted from the electronic medical record system.

We focused on two hallmark features of anti‐LGI1 encephalitis, FBDS and hyponatremia, and stratified patients accordingly. The FBDS subgroup included 24 patients who experienced these seizures; the remaining 23 patients formed the non‐FBDS subgroup. Similarly, 24 patients met criteria for hyponatremia, while 23 did not. An additional control group comprised 25 neurologically healthy individuals without evidence of CNS pathology. Brain MRI was available for 32 anti‐LGI1 patients: 27 demonstrated medial temporal lobe signal abnormalities, and six exhibited basal ganglia involvement.

### 5.2 ^18^F‐FDG PET Imaging Acquisition

^18^F‐ FDG PET/CT scans were performed on a GE Discovery Elite 670 (GE Healthcare, Chicago, IL, USA). All participants fasted for 4–6 h prior to imaging. Each received an intravenous bolus of ^18^F‐FDG (0.10‐0.15 mCi/kg), followed by a 50‐min uptake period in a quiet, dimly lit room. Emission data were then acquired over 10 min and reconstructed using ordered‐subset expectation maximization (OSEM). Subsequently, a low‐dose CT transmission scan (120 kV, 240 mAs) was obtained for attenuation correction. PET images underwent standard corrections for attenuation (based on CT), scatter, random coincidences, radioactive decay, and detector dead‐time.

### 5.3 Pre‐Processing of ^18^F‐FDG PET Data

All PET datasets were preprocessed in SPM12 (Wellcome Department of Cognitive Neurology, London, UK) running under MATLAB R2020a [50]. First, individual PET volumes were normalized to MNI space using the DARTEL algorithm [51, 52] and resampled to 2 × 2 × 2 mm^3^ voxels. The normalized images were spatially smoothed with an 8‐mm full‐width at half‐maximum Gaussian kernel. Finally, to control for inter‐subject variability in global tracer uptake, SUVR maps were generated by dividing voxel‐wise SUVs by an unbiased reference region derived iteratively as described by Nie et al. [53].

### 5.4 Voxel‐Wise Statistical Analysis of ^18^F‐FDG PET Images

Voxel‐wise statistical analyses were conducted in SPM12 using a general linear model framework. First, we compared acute‐phase anti‐LGI1 encephalitis patients (*n* =  47) to healthy controls (*n* =  25) via a two‐sample *t*‐test. Significant clusters of altered metabolism were identified at a voxel‐level threshold of *p* < 0.001, with cluster‐level correction for multiple comparisons based on Gaussian random field theory at *p* < 0.05.

Next, we defined three seed regions of interest, HIP, HYP, and BG, based on the initial group comparison. For each seed, we performed a whole‐brain voxel‐wise linear regression of SUVR against the mean uptake in that ROI. Regions exhibiting significant covariance with each seed were determined using the same statistical thresholds (*p* < 0.001 voxel‐wise; GRF‐corrected cluster *p* < 0.05).

Finally, to explore clinical correlates, we stratified patients into subgroups by the presence or absence of FBDS and by hyponatremia status. Within each stratification, we reran two‐sample *t*‐tests on the SUVR maps to identify subgroup‐specific metabolic differences. Again, significant effects were reported at *p* < 0.001 (voxel‐wise) with cluster‐level GRF correction at *p* < 0.05, ensuring that all findings reflect robust, clinically relevant alterations in brain metabolism.

### 5.5 Construction of LGI1‐Specific Related Pattern

In order to establish the characteristic distribution pattern of brain metabolism in patients with acute stage of anti‐LGI1 encephalitis, we used the Scaled Subprofile Modeling (SSM) model [54] (Figure S3) to establish and verify the LGI1‐specific related pattern based on the scanvp toolkit (version 6.1), employing a fivefold cross‐validation scheme. In each fold, 20 anti‐LGI1 encephalitis patients (LGI1PC) and 20 age‐matched healthy volunteers (NCPC) were randomly assigned to the training set, while the remaining 27 patients (LGI1PV) and five controls (NCPV) comprised the test set. To reduce over‐ and under‐fitting, this split, and the subsequent pattern derivation, was repeated 10 times.

The voxel intensities of each masked SUV image in both LGI1_PC_ and NC_PC_ subgroups were initially vectorized and concatenated to form an M × N data matrix ***P*** ((where *M* is the number of scans and *N* the number of voxels). The matrix ***P*** was then logarithmically transformed to separates essentially meaningless multiplicative scaling effects into additive components. Then, the log *P* were "row‐centered" with respect to its whole‐brain mean log value of each individual, and further "column‐centered" with respect to the group mean log value. Therefore, the resulting doubly centered data could be defined as the individual residual profile (IRP) matric in Equation (1).

(1) $$\;\mathrm{IR}P_{\mathrm{IV}}=\;\mathrm{Log}\;P_{\mathrm{IV}\;}-\mathrm{mea}n_{\mathrm{individual}}\left(\mathrm{Log}P_{\mathrm{IV}}\right)-\mathrm{GM}P_{V\;}$$

Here, subscripts *I* and *V* denote individual and voxel indices, respectively, and GMP refers to the group mean profile.

By singular value decomposition (SVD) of the covariance matrix of IRP, the voxel value of IRP could be expressed as a sum of the unitized group independent subprofile (GIS*_k_ (k* = 1,2,3, …, 16)) vector weights multiplied by its corresponding individual score as illustrated in Equation (2).

(2) $$\;\mathrm{IR}P_{I\;}=\sum_{k}\mathrm{Scor}e_{kI}\mathrm{GI}S_{k}\;$$

Finally, a significant GIS with the maximum separation between the LGI1_PC_ and NC_PC_, which generated by two‐sample Student's *t*‐test, was determined as the final constructed LGI1‐specific related pattern.

Because **GIS** vectors are orthogonal, the individual score of the *k*th **GIS** could be evaluated as in Equation (3).

(3) $$\mathrm{IR}{P_{I}}^{T}\cdot \;\mathrm{GI}S_{k}=\left(\sum_{k}\mathrm{Scor}e_{kI}\mathrm{GI}{S_{k}}^{T}\right)\;\cdot \;\mathrm{GI}S_{k}=\mathrm{Scor}e_{kI}\;$$

For the pattern validation, the individual score of both LGI1_PV_ and NC_PV_ subgroups were calculated by Equation (3), and further *z*‐transformed. Two‐sample Student's *t*‐test was then performed between the *z*‐scores of LGI1_PV_ and NC_PV_. Statistical significance was defined as a *p* < 0.05.

### 5.6 Associations Between the LGI1‐Specific Metabolic Pattern and Clinical Measures

Pattern expression scores (*z*‐scores) generated by the SSM model were correlated with clinical data using SPSS v25.0. We first assessed normality of continuous variables via the Shapiro–Wilk test. For normally distributed measures (e.g., MoCA), Pearson correlation was applied to evaluate associations with pattern *z*‐scores. Spearman rank correlation was used for non‐normally distributed variables, including age, MMSE, acute‐phase, and remission‐phase CASE scores, as well as for categorical factors such as sex, education level, and mRS. Statistical significance was defined as *p* < 0.05.

### 5.7 Construction of LGI1‐Specific Topological Network

Using the Brainnetome atlas [54], the central brain regions of anti‐LGI1 encephalitis involved in the pattern were further subdivided into finer subregions. The Person's correlations (*R*) of brain metabolism covariance of each pair of sub‐regions were then computed, leading to the establishment of a metabolic covariant network [55].

To compare network topology between patients and controls, we employed a permutation‐based *t*‐test on the difference in connectivity strength (ΔR). In each of 5,000 iterations, all participants were pooled and randomly reassigned into a pseudo‐control group (*n* = 25) and a pseudo‐patient group (remaining subjects). Covariance networks were reconstructed for each resampled grouping, and a permuted ΔR was calculated. Empirical *p*‐values below 0.05 were considered indicative of significant network alterations.

## Author Contributions

Wenping Ma, Fu‐Dong Shi, Lin Ai, and Wangshu Xu contributed to the conception and design of the study. Binbin Nie, Xuan Xu, Wenyue Dong, Leilei yuan, Hengri Cong, Yueta Ma, Huabing Wang, De‐Cai Tian, Linlin Yin, Tian Song, Yanxue Zhao, Guoqiang Chang, TianJie Lyu, and Yun Liu contributed to the acquisition and analysis of data. Binbin Nie, Xuan Xu, and Wenyue Dong contributed to drafting the text or preparing the figures. Wenping Ma, Fu‐Dong Shi, Lin Ai, and Wangshu Xu provided valuable comments on the manuscript and carried out significant revisions. All authors have read and approved the final manuscript.

## Funding

The study was financed by Beijing Natural Science Foundation grant 7232044 and the National Natural Science Foundation of China grant 12175268.

## Ethics Statement

This study was approved by the Ethics Committee of Beijing Tiantan Hospital, Capital Medical University, Beijing, China (KY2023‐249‐02). All participants had informed consent to participate in the study before taking part. This study did not involve any clinical trials, so the clinical trial registration number is not applicable.

## Conflicts of Interest

The authors declare no conflicts of interest.

## Supporting information



Figure S1 **Detailed brain metabolic distribution pattern in patients with anti‐LGI1 encephalitis in acute phase**. A detailed voxel‐based two‐sample t test between anti‐LGI1 encephalitis patients and the controls, generated at a voxel threshold of p<0.001 (corrected for GRF with p<0.05 for cluster size). Hypermetabolized voxels show warm colors, while hypometabolized voxels show cool colors.
Figure S2 **Bar charts illustrating the subregional metabolic differences between anti‐LGI1 encephalitis patients and controls**. Subregional metabolism of the DLPFC(A). Subregional metabolism of the basal ganglia (B). Subregional metabolism of the cingulate cortex(C). DLPFC, dorsolateral prefrontal cortex; LGI1, leucine‐rich glioma inactivated 1; NC, normal control. *: p<0.05; **: p<0.01;***: p<0.001
Figure S3 **The calculation method flow chart of establishing the SSM model**
Table S 1 Information about patients with anti‐LGI1 encephalitis in acute phase

## Data Availability

The datasets generated during and/or analyzed during the current study are available from the corresponding author on reasonable request.
