# Supplementary material for: 18F‐ FDG PET Reveals a Nucleus Accumbens‐Centered Metabolic Network Correlating With Clinical Severity in Anti‐LGI1 Encephalitis
Source: MedComm (2020). 2025 Dec 14;6(12):e70544. doi: 10.1002/mco2.70544 (PMC12701962; doi:10.1002/mco2.70544)
Supplement: Supplementary file 1 — Figure S1 Detailed brain metabolic distribution pattern in patients with anti‐LGI1 encephalitis in acute phase. A detailed voxel‐based two‐sample t test between anti‐LGI1 encephalitis patients and the controls, generated at a voxel threshold of p<0.001 (corrected for GRF with p<0.05 for cluster size). Hypermetabolized voxels show warm colors, while hypometabolized voxels show cool colors. Figure S2 Bar charts illustrating the subregional metabolic differences between anti‐LGI1 encephalitis patients and controls. Subregional metabolism of the DLPFC(A). Subregional metabolism of the basal ganglia (B). Subregional metabolism of the cingulate cortex(C). DLPFC, dorsolateral prefrontal cortex; LGI1, leucine‐rich glioma inactivated 1; NC, normal control. *: p<0.05; **: p<0.01;***: p<0.001 Figure S3 The calculation method flow chart of establishing the SSM model Table S 1 Information about patients with anti‐LGI1 encephalitis in acute phase [file MCO2-6-e70544-s001.docx]

**^18^F-** **FDG PET reveals a nucleus accumbens-centered metabolic network correlating with clinical severity in anti‑LGI1 encephalitis**

Binbin Nie, PhD^1,2^#, Xuan Xu, MM^3^#, Wenyue Dong, MM^4^#, Leilei yuan, MD^5^, Hengri Cong, MD^3^, Yueta Ma, MD^3^, Huabing Wang, MD^3^, De-Cai Tian, MD, PhD^3^, Linlin Yin, MD^3^, Tian Song, MD^3^, Yanxue Zhao, MD^3^, Guoqiang Chang, MD^6^, TianJie Lyu, MD ^3^, Yun Liu, MD^3^, Wenping Ma, MD^7*^, Fu-Dong Shi, MD, PhD^3^*, Lin Ai, MD^5^*, Wangshu Xu, MD^3^*

^1^Beijing Engineering Research Center of Radiographic Techniques and Equipment, Institute of High Energy Physics, Chinese Academy of Sciences, Beijing 100049, China

^2^School of Nuclear Science and Technology, University of Chinese Academy of Sciences, Beijing 100049, China

^3^Department of Neurology, Beijing Tiantan Hospital, Capital Medical University, Beijing 100070, China

^4^Peking University Third Hospital, Beijing 100191, China

^5^Department of Nuclear Medicine, Beijing Tiantan Hospital, Capital Medical University, Beijing 100070, China

^6^Department of Neurology, Tianjin Medical University General Hospital, Tianjin 300052, China

^7^Department of Neurosurgery, Beijing Children’s Hospital, Capital Medical University, National Center for Children’s Health, Beijing 100045, China

#These authors contributed equally: Binbin Nie, Xuan Xu, Wenyue Dong

*Equally as corresponding authors: Wenping Ma^7^, Fu-Dong Shi^3^, Lin Ai^5^, Wangshu Xu^3^

Email: mawenping@bjmu.edu.cn; fshi@tmu.edu.cn; ailin@bjtth.org; [xuwangshu@mail.ccmu.edu.cn](mailto:xuwangshu@mail.ccmu.edu.cn)


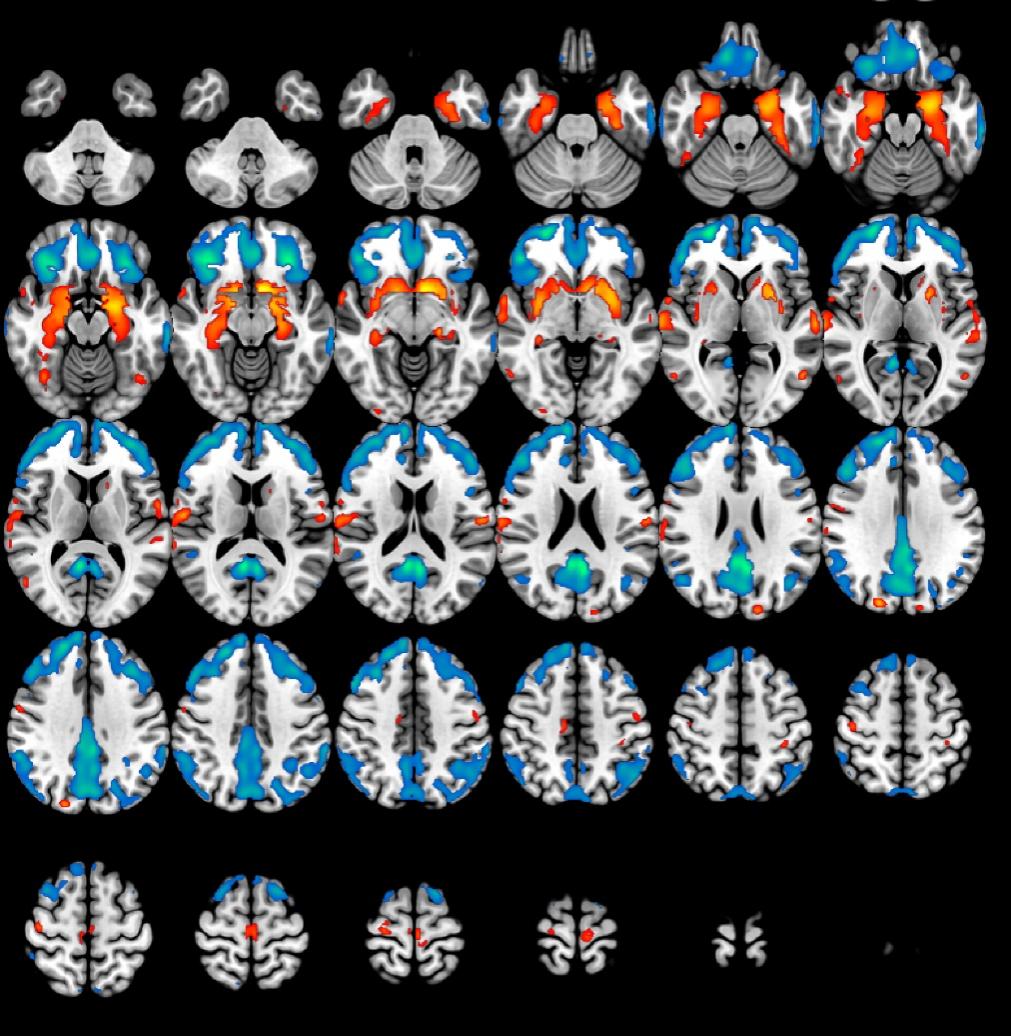


Figure S1 **Detailed brain metabolic distribution pattern in patients with anti-LGI1 encephalitis in acute phase.** A detailed voxel-based two-sample t test between anti-LGI1 encephalitis patients and the controls, generated at a voxel threshold of p<0.001 (corrected for GRF with p<0.05 for cluster size). Hypermetabolized voxels show warm colors, while hypometabolized voxels show cool colors.


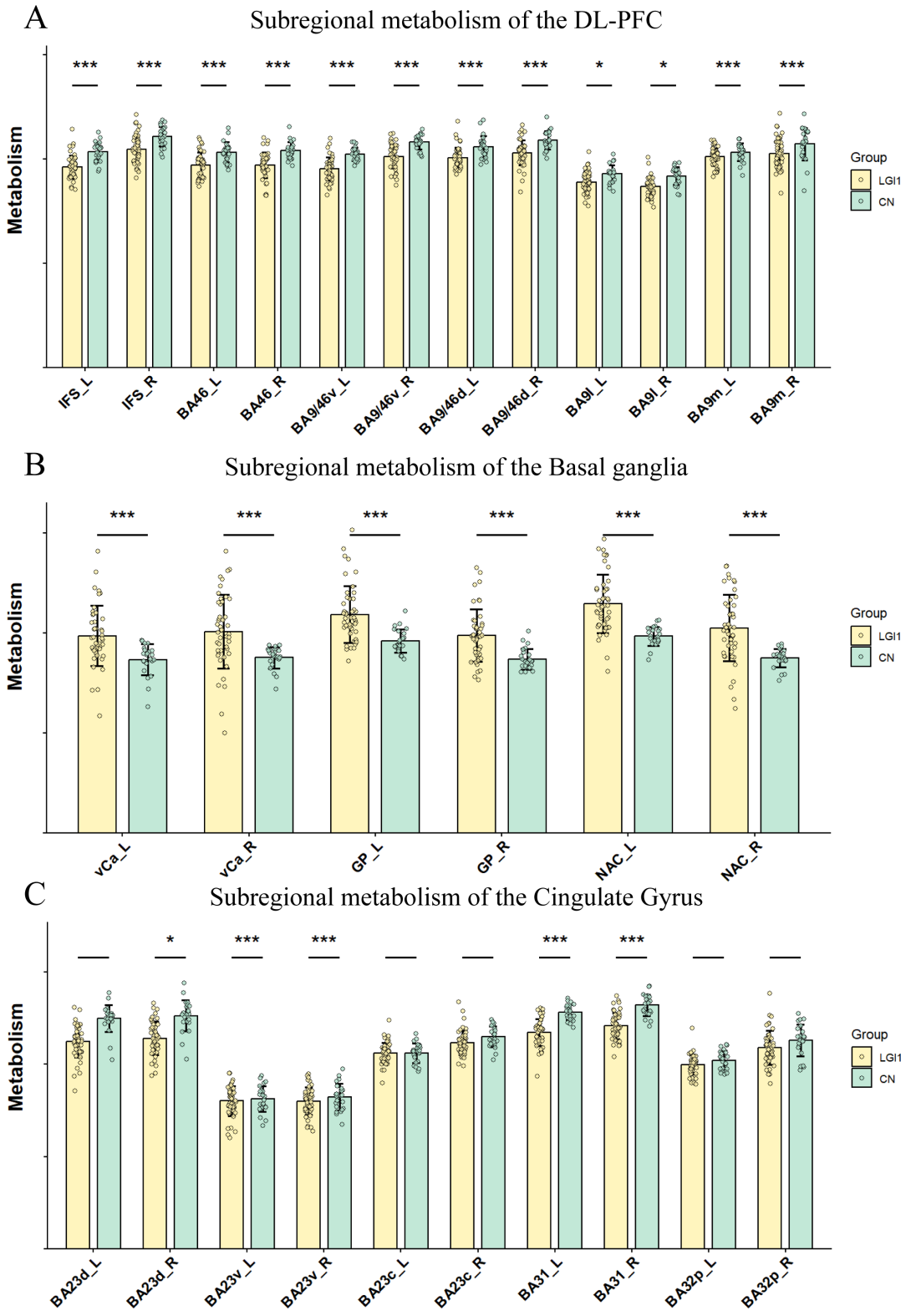


Figure S2 **Bar charts illustrating the subregional metabolic differences between anti-LGI1 encephalitis patients and controls.** Subregional metabolism of the DLPFC(A). Subregional metabolism of the basal ganglia (B). Subregional metabolism of the cingulate cortex(C). DLPFC, dorsolateral prefrontal cortex; LGI1, leucine-rich glioma inactivated 1; NC, normal control. *: p<0.05; **: p<0.01;***: p<0.001


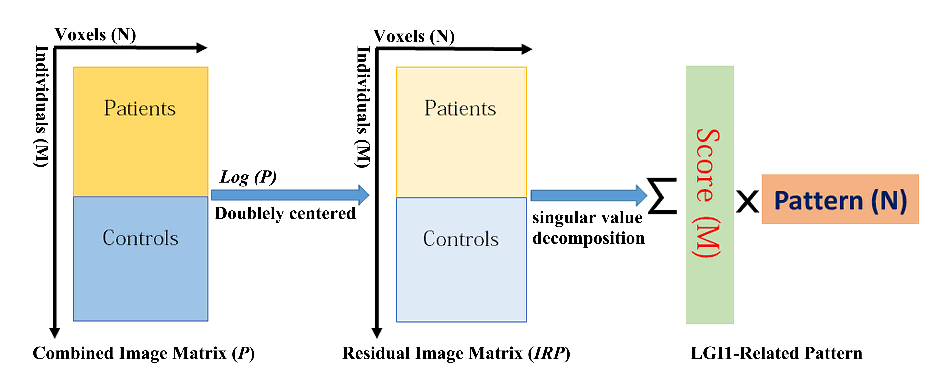


Figure S3 **The calculation method flow chart of establishing the SSM model**

Table S 1 Information about patients with anti-LGI1 encephalitis in acute phase

|  | Sex | Age | MoCA | MMSE | mRS | CASE | z-score pattern | Time when the latest symptoms appeared | Time to complete the ^18^F FDG PET scans | Time interval from the onset of the latest symptoms to the completion of the scan(days) | Treatments | Outcome | mRS (at discharge) | CASE (remission) |  |
| --- | --- | --- | --- | --- | --- | --- | --- | --- | --- | --- | --- | --- | --- | --- | --- |
| Patient 1 | Male | 65 | 27 | 28 | 1 | 4 | 1.705 | 2021/5/15 | 2021/7/20 | 66 | IVGC, IVIG | Improvement | 1 | / |  |
| Patient 2 | Male | 52 | 20 | 23 | 2 | 8 | 0.649 | 2021/2/10 | 2021/4/12 | 61 | IVGC, IVIG | Improvement | 1 | 1 |  |
| Patient 3 | Male | 44 | 15 | 13 | 2 | 3 | 1.713 | 2020/8/21 | 2020/9/4 | 14 | IVGC, IVIG | Improvement | 1 | 1 |  |
| Patient 4 | Male | 69 | 16 | 15 | 2 | 4 | 2.737 | 2020/6/26 | 2020/7/15 | 19 | IVGC, IVIG | Improvement | 1 | / |  |
| Patient 5 | Male | 69 | 17 | 16 | 3 | 5 | 1.047 | 2019/10/24 | 2019/11/19 | 26 | IVGC, IVIG | Improvement | 2 | 5 |  |
| Patient 6 | Female | 38 | 26 | 29 | 2 | 3 | 2.095 | 2021/12/13 | 2022/1/6 | 24 | IVGC, IVIG | Improvement | 2 | 1 |  |
| Patient 7 | Male | 63 | 12 | 13 | 3 | 4 | 0.942 | 2023/3/13 | 2023/5/10 | 58 | IVGC, IVIG | Improvement | 2 | / |  |
| Patient 8 | Male | 32 | 27 | 30 | 1 | 2 | 0.763 | 2021/6/3 | 2021/7/9 | 36 | No immunotherapy | Improvement | 0 | 1 |  |
| Patient 9 | Female | 43 | / | / | 1 | 3 | 0.51 | 2021/5/31 | 2021/7/19 | 49 | IVGC, IVIG | Improvement | 1 | 1 |  |
| Patient 10 | Female | 46 | 25 | 30 | 2 | 2 | 1.609 | 2022/10/1 | 2022/12/30 | 90 | IVGC, IVIG | Improvement | 1 | 2 |  |
| Patient 11 | Male | 64 | / | / | 3 | 3 | 1.666 | 2018/4/17 | 2018/7/3 | 77 | IVIG | Improvement | 2 | 0 |  |
| Patient 12 | Male | 74 | / | / | 3 | 3 | 1.899 | 2017/6/16 | 2017/7/11 | 25 | IVGC, IVIG | Improvement | 2 | / |  |
| Patient 13 | Female | 65 | / | / | 3 | 5 | 1.089 | 2017/8/15 | 2018/1/17 | 155 | IVGC | Improvement | 2 | / |  |
| Patient 14 | Male | 53 | 22 | 29 | 2 | 2 | -0.086 | 2021/3/9 | 2021/4/13 | 35 | IVIG | Improvement | 1 | 1 |  |
| Patient 15 | Male | 64 | 23 | 25 | 1 | 4 | 1.986 | 2019/11/12 | 2019/12/17 | 35 | IVIG | Improvement | 1 | / |  |
| Patient 16 | Female | 57 | 5 | 13 | 4 | 9 | 2.082 | 2020/6/22 | 2020/7/3 | 11 | IVGC, IVIG, MMF | Progression | 5 | / |  |
| Patient 17 | Female | 30 | / | / | 1 | 5 | 1.642 | 2020/6/14 | 2020/7/8 | 24 | IVIG | Improvement | 1 | 0 |  |
| Patient 18 | Male | 57 | 18 | 29 | 2 | 6 | 1.383 | 2020/5/15 | 2020/6/12 | 28 | IVGC, IVIG, MMF | Improvement | 1 | 1 |  |
| Patient 19 | Female | 53 | 26 | 30 | 1 | 2 | 0 | 2020/3/25 | 2020/6/17 | 84 | No immunotherapy | Improvement | 1 | 1 |  |
| Patient 20 | Male | 38 | 22 | 27 | 2 | 3 | 1.258 | 2020/5/28 | 2020/6/10 | 13 | IVGC, IVIG | Improvement | 2 | 1 |  |
| Patient 21 | Female | 72 | 22 | 24 | 2 | 5 | 0.653 | 2020/5/12 | 2020/6/4 | 23 | IVIG | Improvement | 1 | 1 |  |
| Patient 22 | Male | 71 | 23 | 25 | 1 | 6 | 0.978 | 2022/4/9 | 2022/5/12 | 33 | IVIG | Improvement | 1 | 2 |  |
| Patient 23 | Female | 47 | 24 | 28 | 2 | 3 | 0.461 | 2020/10/20 | 2020/11/25 | 36 | IVIG | Improvement | 1 | 3 |  |
| Patient 24 | Male | 61 | 17 | 27 | 2 | 3 | 1.491 | 2020/7/18 | 2020/8/7 | 20 | IVGC | Improvement | 1 | 0 |  |
| Patient 25 | Male | 66 | 20 | 27 | 3 | 2 | 0.771 | 2021/4/21 | 2021/6/24 | 64 | IVGC | Improvement | 2 | 1 |  |
| Patient 26 | Female | 31 | 16 | 22 | 3 | 3 | 1.158 | 2020/12/26 | 2021/6/7 | 163 | IVGC, IVIG | Improvement | 2 | / |  |
| Patient 27 | Female | 56 | 25 | 28 | 2 | 5 | 2.089 | 2021/10/17 | 2021/11/25 | 39 | IVGC, IVIG | Improvement | 1 | 1 |  |
| Patient 28 | Female | 55 | / | / | 4 | 2 | 1.232 | 2021/10/16 | 2021/11/9 | 24 | IVGC | Improvement | 1 | 1 |  |
| Patient 29 | Female | 61 | 23 | 29 | 2 | 2 | 1.783 | 2021/9/18 | 2021/10/9 | 21 | IVGC, IVIG | Improvement | 1 | 0 |  |
| Patient 30 | Female | 59 | 12 | 23 | 2 | 3 | 1.936 | 2022/6/25 | 2022/7/22 | 27 | IVGC, IVIG | Improvement | 1 | / |  |
| Patient 31 | Male | 62 | / | / | 1 | 2 | 1.762 | 2022/3/17 | 2022/4/11 | 25 | IVIG | Improvement | 1 | 1 |  |
| Patient 32 | Female | 40 | 26 | 30 | 3 | 3 | 0.796 | 2022/5/6 | 2022/6/14 | 39 | IVGC, IVIG | Improvement | 1 | 2 |  |
| Patient 33 | Female | 71 | 21 | 27 | 4 | 3 | 2.44 | 2022/5/1 | 2022/5/24 | 23 | IVGC | Improvement | 2 | / |  |
| Patient 34 | Female | 77 | 18 | 28 | 2 | 2 | 0.913 | 2022/4/29 | 2022/5/23 | 24 | IVIG | Improvement | 1 | 1 |  |
| Patient 35 | Female | 30 | 21 | 21 | 1 | 2 | 1.513 | 2022/3/28 | 2022/4/14 | 17 | IVIG | Improvement | 1 | 0 |  |
| Patient 36 | Male | 76 | / | / | 3 | 4 | 2.073 | 2021/12/18 | 2022/1/24 | 37 | IVGC, IVIG | Improvement | 1 | 2 |  |
| Patient 37 | Female | 68 | 12 | 16 | 4 | 5 | 1.672 | 2022/10/19 | 2022/12/23 | 65 | IVGC, IVIG | Improvement | 2 | 1 |  |
| Patient 38 | Male | 55 | 18 | 24 | 2 | 7 | 1.832 | 2022/4/17 | 2022/5/19 | 32 | IVGC, IVIG | Improvement | 2 | 2 |  |
| Patient 39 | Female | 66 | 17 | 22 | 2 | 4 | 1.74 | 2022/5/3 | 2022/8/15 | 104 | IVIG | Improvement | 1 | 1 |  |
| Patient 40 | Male | 57 | 22 | 26 | 3 | 4 | 2.002 | 2023/4/1 | 2023/6/19 | 79 | IVGC, IVIG | Improvement | 2 | 2 |  |
| Patient 41 | Female | 21 | 23 | 24 | 4 | 3 | 2.842 | 2022/8/26 | 2022/9/29 | 34 | IVGC, IVIG | Improvement | 2 | 3 |  |
| Patient 42 | Female | 68 | 23 | 28 | 1 | 1 | -0.208 | 2023/7/30 | 2023/9/1 | 33 | IVGC, IVIG | Improvement | 1 | 1 |  |
| Patient 43 | Male | 67 | 12 | 20 | 3 | 4 | 3.273 | 2022/2/9 | 2022/3/1 | 20 | IVIG | Improvement | 3 | / |  |
| Patient 44 | Male | 66 | 19 | 18 | 2 | 2 | 1.185 | 2023/12/2 | 2023/12/20 | 18 | IVGC, MMF | Improvement | 1 | / |  |
| Patient 45 | Male | 58 | / | / | 2 | 3 | 1.275 | 2023/10/17 | 2023/12/22 | 66 | IVGC, IVIG | Improvement | 1 | 2 |  |
| Patient 46 | Male | 52 | 12 | 16 | 3 | 4 | 2.071 | 2023/8/6 | 2023/8/31 | 25 | IVIG | Improvement | 3 | 2 |  |
| Patient 47 | Male | 79 | / | / | 4 | 8 | 2.202 | 2023/8/11 | 2023/10/16 | 66 | IVIG | Improvement | 4 | 8 |  |
| MoCA, Montreal Cognitive Assessment; MMSE, Mini-Mental State Examination; mRS, Modified Rankin Scale; CASE, Clinical Assessment Scale for Autoimmune Encephalitis; IVGC, intravenous glucocorticoid; IVIG, Intravenous immunoglobulin; MMF, mycophenolate mofetil. | | | | | | | | | | | | | | |  |
